# Establishing a 3-Tesla Magnetic Resonance Imaging Method for Assessing Diffuse Axonal Brain Injury in Rats

**DOI:** 10.3390/ijms25084234

**Published:** 2024-04-11

**Authors:** Dmitry Frank, Benjamin F. Gruenbaum, Vladislav Zvenigorodsky, Ilan Shelef, Anna Oleshko, Frederic Matalon, Beatris Tsafarov, Alexander Zlotnik, Amit Frenkel, Matthew Boyko

**Affiliations:** 1 Department of Anesthesiology and Critical Care, Soroka University Medical Center, Ben-Gurion University of the Negev, Beer-Sheva 84101, Israel; frdima16@gmail.com (D.F.);; 2 Department of Anesthesiology and Perioperative Medicine, Mayo Clinic, Jacksonville, FL 32224, USA; 3 Department of Radiology, Soroka University Medical Center and the Faculty of Health Sciences, Ben-Gurion University of the Negev, Beer-Sheva 84101, Israel; zvenigorodsky@gmail.com (V.Z.); shelef@bgu.ac.il (I.S.); 4 Department of Biology and Methods of Teaching Biology, A. S. Makarenko Sumy State Pedagogical University, 40002 Sumy, Ukraine; 5 Department of Histology, Soroka University Medical Center and the Faculty of Health Sciences, Ben-Gurion University of the Negev, Beer-Sheva 84101, Israel; 6 Department of Emergency Medicine Recanati School for Community Health Professions, Ben-Gurion University of the Negev, Beer-Sheva 84101, Israel; frenkela@bgu.ac.il

**Keywords:** 3-Tesla, diffuse axonal brain injury, magnetic resonance imaging, rats, traumatic brain injury

## Abstract

Traumatic brain injury (TBI) significantly contributes to death and disability worldwide. However, treatment options remain limited. Here, we focus on a specific pathology of TBI, diffuse axonal brain injury (DABI), which describes the process of the tearing of nerve fibers in the brain after blunt injury. Most protocols to study DABI do not incorporate a specific model for that type of pathology, limiting their ability to identify mechanisms and comorbidities of DABI. In this study, we developed a magnetic resonance imaging (MRI) protocol for DABI in a rat model using a 3-T clinical scanner. We compared the neuroimaging outcomes with histologic and neurologic assessments. In a sample size of 10 rats in the sham group and 10 rats in the DABI group, we established neurological severity scores before the intervention and at 48 h following DABI induction. After the neurological evaluation after DABI, all rats underwent MRI scans and were subsequently euthanized for histological evaluation. As expected, the neurological assessment showed a high sensitivity for DABI lesions indicated using the β-APP marker. Surprisingly, however, we found that the MRI method had greater sensitivity in assessing DABI lesions compared to histological methods. Out of the five MRI parameters with pathological changes in the DABI model, we found significant changes compared to sham rats in three parameters, and, as shown using comparative tests with other models, MRI was the most sensitive parameter, being even more sensitive than histology. We anticipate that this DABI protocol will have a significant impact on future TBI and DABI studies, advancing research on treatments specifically targeted towards improving patient quality of life and long-term outcomes.

## 1. Introduction

Traumatic brain injury (TBI) has a significant impact globally [1] as a leading contributor to death and disability [2,3]. Traumatic brain injury constitutes up to one-third of all accidental deaths, and the majority of deaths are due to trauma in the hospital setting [4,5,6,7].

Diffuse axonal injury (DAI) is a common type of traumatic brain injury, occurring in approximately 40–50% of all TBI patients. Following TBI, sufferers can continue to be affected by lifelong disability [8]. Despite impressive efforts to advance treatment modalities for both TBI itself and its secondary conditions, treatment efficacy is still limited [9,10]. Recent data suggests the mortality rate of DAI is 42–62% [1,2].

Diffuse axonal brain injury (DABI) is one of the most common and important pathologic features of TBI [11], occurring in almost 50% of all patients suffering from TBI. This type of traumatic brain injury is caused by the tearing of the brain’s long connective nerve fibers when the brain moves inside the skull due to a blunt injury to the head [12,13,14,15,16]. The forceful tearing of axons in the brain disrupts communication between nerves, leading to physical and cognitive impairment. This injury can result in a coma and damage to various brain regions, causing a spectrum of neurological deficits encompassing cognitive impairments, confusion, a shortened attention span, memory issues, and amnesia, and can affect social reintegration, productivity, and quality of life [12]. The severity of impairments depends on the function of the affected nerves.

Due to the ethical concerns and practical challenges of studying TBI in humans, diverse animal models have been developed to help understand its pathophysiology. While there are many animal models of TBI, the disorder is complex and influenced by multiple factors. Thus, research models attempt to replicate the complex dynamics of TBI through several methods: focal impact, diffuse impact, blast injuries, and non-impact injuries induced by rotational acceleration.

While focal models are useful for understanding the direct impact and subsequent biological response, diffuse models mimic the widespread neural damage that more closely resembles the multifaceted nature of human TBI [17].

Although DABI always accompanies TBI to some degree, the pathophysiology of DABI is fundamentally different from TBI. Thus, in order to effectively study the pathophysiology of DABI and test treatment strategies, a suitable model in which DABI is isolated from TBI must be used. Although DABI is a distinct pathology that always accompanies TBI and requires similarly careful study, it has received little attention, and when it is studied, it is mostly in unspecialized animal models [17,18,19,20].

Magnetic resonance imaging (MRI) is a necessary technique for examining the brain, assisting with the analysis of stroke and TBI without exposing people or animals to harmful radiation. MRI is widely known to be non-invasive and very adaptable. In addition, MRI is used to produce a superior level of soft tissue contrast, along with high temporal and spatial resolutions, making it a suitable technique for models with small animals [21]. Most animal models of stroke and TBI published previously have had the goal of finding the extent and location of brain injury after a stroke and TBI. These protocols involve animal sacrifice, which, besides its ethical concerns, also precludes the option of conducting later studies with the same study group. These failures have, therefore, prompted the increased use of clinical MR scanners to image small animals. Recently, MRI techniques have been used in experimental studies using animal models to monitor the neurologic outcomes of stroke and TBI, such as brain edema, lesion volume, BBB destruction, and cerebral blood flow, and to evaluate different treatment regimens [22,23,24,25,26,27].

The MRI method is popular among studies using rat models of TBI, and a PubMed search found 685 scientific publications ((rat) OR (mice)) AND (traumatic brain injury) AND (MRI). However, a similar search using the DABI model with the terms ((rat) OR (mice)) AND (diffuse axonal injury) AND (rotation) AND (MRI) yielded only three results. We believe that the main reasons that this important topic is understudied involve the lack of a validated and reliable model specialized for DABI and the lack of reliable neuroimaging techniques to assess DABI. The purpose of this study was to develop an MRI protocol for DABI and to compare the neuroimaging outcomes with histologic and neurologic assessments. In this paper, we focused on the study of neuroimaging markers using a rotating DABI model and utilized a clinical scanner to establish an MRI protocol, hypothesizing that the MRI protocol would be sufficient in attaining an understanding of DABI presentation. We believe that this protocol will assist in the deeper understanding of DABI and TBI, more generally, with the eventual goal of safe and effective brain trauma treatments.

## 2. Results

### 2.1. Neurological Severity Score

Because the neurological scale corresponds to data that meet the definition of an ordinal scale, the appropriate test for this type of data when comparing the means in two groups is the two-tailed Mann–Whitney U test, implemented here. The data appeared as the median ± inner quartile range. The effect size, r, was calculated by dividing Z by the square root of N (r = Z/√N). The data were measured as a count and expressed as median and 25–75 percentile range.

The NSS for each group 48 h after TBI appears in Table 1. As anticipated, there was no difference between the groups at baseline before the intervention. The sham-operated group did not have any neurological deficit at 48 h (NSS-0). Compared to the sham-operated group, the NSS at 48 h was significantly greater after DABI [1(1–2.5) vs. 0(0–0), U = 13.5, *p* < 0.01, r = 0.69] according to the Mann–Whitney U test.

### 2.2. Analysis of the Immunohistochemistry for β-APP

Because the number of β-APP-positive axons per mm^2^ corresponds to data that meet the definition of an interval scale, the appropriate test for this type of data when comparing the means in two groups is the two-tailed Student’s *t*-test. The effect sizes were represented by Cohen’s d, which indicates the size of the treatment effect relative to the within-group variability of the scores. The data were measured as the counts of the β-APP-positive axons of the DABI rats vs. the control group and expressed as the mean ± SD.

**Figure 1:**
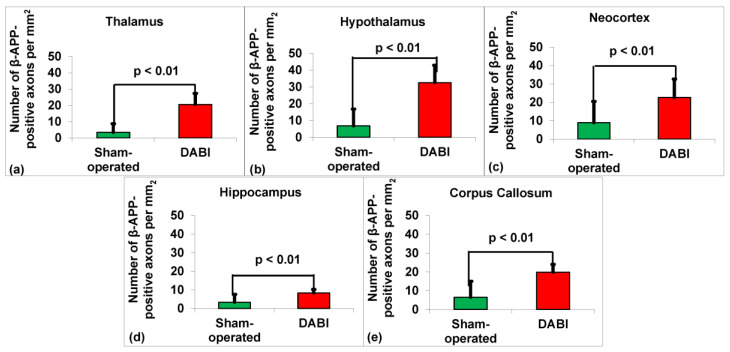
Graph showing the density of β-APP-positive axons in the study groups in different brain areas. (**a**) Thalamus. (**b**) Hypothalamus. (**c**) Neocortex. (**d**) Hippocampus. (**e**) Corpus callosum.

Student’s *t*-test revealed a statistically significant difference in the number of β-APP-positive axons per mm^2^ in 10 DABI rats compared to the 10 sham-operated rats in the thalamus 20.5 ± 6.9 vs. 3.4 ± 5.3, t(18)= 6.2, *p* < 0.01), d = 2.8 (see Figure 1a); hypothalamus 32.5 ± 10.4 vs. 6.8 ± 10, t(18) = 5.6, *p* < 0.01), d = 2.5 (see Figure 1b); neocortex 22.6 ± 10 vs. 8.1 ± 11.6, t(18)= 2.8, *p* < 0.01), d = 1.3 (see Figure 1c); hippocampus 8.3 ± 2 vs. 3.3 ± 4.3, t(12.6) = 3.4, *p* < 0.01), d = 1.5 (see Figure 1d); and corpus callosum 19.8 ± 4.2 vs. 6.5 ± 8.5, t(13.1) = 4.4, *p* < 0.01), d = 2. (see Figure 1e).

### 2.3. Analysis of the Neuroimaging Outcomes from MRI

Because the MRI outcomes (fractional anisotropy, relative anisotropy, radial diffusivity, axial diffusivity, and mean diffusivity) correspond to data that meet the definition of an interval scale, the appropriate test for this type of data when comparing the means in two groups is the two-tailed Student’s *t*-test. The effect sizes were represented by Cohen’s d, which indicates the size of the treatment effect relative to the within-group variability of the scores. The data were measured as the mm^2^/s or counts of the DABI rats vs. the control group and expressed as the mean ± SD.

**Figure 2:**
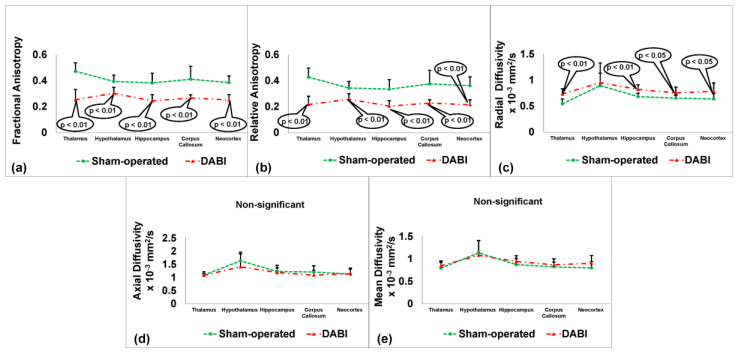
MRI-determined outcome parameters 48 h after DABI. (**a**) Fractional anisotropy. (**b**) Relative anisotropy. (**c**) Radial diffusivity. (**d**) Axial diffusivity. (**e**) Mean diffusivity.

The analysis using the Student’s *t*-test showed a statistically significant difference between the 10 DABI rats and the 10 sham-operated rats in several areas. Fractional anisotropy differences (Figure 2a) were significant in the thalamus (0.25 ± 0.08 vs. 0.47 ± 0.07, t(18) = 6.8, *p* < 0.01, d = 2.9), hypothalamus (0.3 ± 0.05 vs. 0.39 ± 0.05, t(18) = 4.3, *p* < 0.01, d = 1.8), neocortex (0.25 ± 0.04 vs. 0.39 ± 0.05, t(18) = 6.4, *p* < 0.01, d = 3.1), hippocampus (0.24 ± 0.05 vs. 0.38 ± 0.08, t(18) = 4.9, *p* < 0.01, d = 2.1), and corpus callosum (0.27 ± 0.02 vs. 0.41 ± 0.1, t(10) = 4.43, *p* < 0.01, d = 1.9). Relative anisotropy differences (Figure 2b) were significant in the thalamus (0.22 ± 0.06 vs. 0.43 ± 0.07, t(18) = 7.1, *p* < 0.01, d = 3.2), hypothalamus (0.26 ± 0.04 vs. 0.34 ± 0.05, t(18) = 4.1, *p* < 0.01, d = 1.8), neocortex (0.21 ± 0.04 vs. 0.36 ± 0.07, t(18) = 6, *p* < 0.01, d = 2.6), hippocampus (0.2 ± 0.04 vs. 0.33 ± 0.07, t(18) = 4.9, *p* < 0.01, d = 2.3), and corpus callosum (0.23 ± 0.03 vs. 0.38 ± 0.1, t(10.1) = 4.37, *p* < 0.01, d = 2). In the radial diffusivity measures (Figure 2c), significant differences were found in the thalamus (0.73 ± 0.1 vs. 0.54 ± 0.09, t(18) = 4.35, *p* < 0.01, d = 2), neocortex (0.78 ± 0.16 vs. 0.64 ± 0.1, t(18) = 2.5, *p* < 0.05, d = 1.1), hippocampus (0.82 ± 0.09 vs. 0.68 ± 0.07, t(18) = 4.1, *p* < 0.01, d = 1.7), and corpus callosum (0.75 ± 0.11 vs. 0.65 ± 0.06, t(18) = 2.6, *p* < 0.05, d = 1.1). These measurements are presented as mean ± SD in mm^2^/s. No significant differences were observed in the mean and axial diffusivity (Figure 2d,e).

### 2.4. Correlation Comparisons between the MRI Parameters in the DABI Rats

The correlations were determined between the different MRI parameters (see Table 2).

### 2.5. Sensitivity Analysis

To evaluate the sensitivity of the DABI model based on the mean and standard deviation data, we calculated the minimum number of rats for the sham and DABI groups with a power of 0.8 alpha 0.05 for the NSS, MRI, and histological outcomes (see Table 3).

In general, the results shown in Table 3 show that the highest sensitivity of the diagnostic method in the DABI model was seen in MRI when measuring the FA in the neocortex area and the RA in the thalamus areas, where the group size was five rats in order to register a significant difference between the experimental groups. A similar result was achieved in a group of six rats, with histology in the area of the thalamus and hypothalamus. The neurological scale showed less sensitivity because a group size of eight animals was required to achieve a significant difference.

### 2.6. Sensitivity Analysis for Different Experimental Models

To compare the sensitivity of the outcomes among the different experimental models, we calculated the minimum number of animals per group to evaluate the neurological, histological, and MRI outcomes between the models (see Table 4). 

For the histological outcomes, the DABI model (n = 6 for immunohistochemistry for β-APP in the thalamus) showed higher sensitivity compared with the SAH model (n = 20 for brain edema) and TBI model (n = 9 for BBB) but was less sensitive than a stroke model (n = 4 for brain edema).

For MRI, the DABI model (n = 5 in fractional anisotropy and relative anisotropy) showed higher sensitivity compared with the stroke (n = 6 in ADC) and TBI (n = 8 in ADC) models. An MRI assessment for SAH was not performed.

For the NSS, the DABI model (n = 8) showed the least sensitivity compared with the stroke (n = 4), TBI (n = 7), and SAH (n = 2) models. It is worth noting that different brain injury models use different tests to assess neurological deficits.

Of the five MRI parameters that could potentially show pathological changes in the DABI model (MD, AD, RD, RA, and FA), we found significant changes compared to the sham rats in three parameters (RD, RA, and FA), and as shown using the comparative tests with other models (see Table 3 and Table 4), MRI was the most sensitive parameter, being even more sensitive than histology.

The comparison of the MRI parameters showed the strongest correlation in the same areas when measured using different methods (see Table 2, marked in color). The highest correlation was in the hypothalamus, and the lowest correlation was in the hippocampus and corpus callosum (see Table 2).

## 3. Discussion

Despite the well-documented connection between DABI and TBI, there are very few scientific studies examining the pathophysiology of DABI in DABI-specific models. A PubMed search yielded 10,715 publications related to studies of TBI in rat models using the search words ((rat) OR (mice)) AND (traumatic brain injury)). A query made in PubMed to identify studies investigating DABI using the search terms (rat) OR (mice)) AND (diffuse axonal injury) found a total of 462 studies addressing DABI. The papers investigating DABI have overwhelmingly used impact-related TBI models [31,32], such as Marmarou [33], without applying specialized models established for DABI [34], such as a rotation model [35]. A search in the PubMed database using the search terms ((rat) OR (mice)) AND (diffuse axonal injury) AND (rotation) only identified 40 publications that used a specialized model for DABI induction.

We have shown the importance of specialized models of TBI and stroke in previous publications. We established a rotational model for the induction of isolated DABI and evaluated it using histological and neurobehavioral markers [34]. We also developed a protocol for a neuroimaging assessment of stroke [23] and TBI [22] outcomes in rats using the 3-T clinical scanner.

Our results show that DABI rats did not differ in their neurological assessments from sham rats in the categories of mobility, hemiplegia, reflexes, and behavior. We recorded differences between the experimental groups only in the categories of beam walking and beam balance between the groups’ stages. Thus, it appears that only the beam walking and beam balance categories are useful for neurological assessments in the DABI model. Overall, the extent of neurological impairment was significantly lower in rats using the DABI model (see Table 1) compared to the TBI, stroke, and subarachnoid hemorrhage (SAH) models we have established previously [24,25,27,28,29,30].

To compare the sensitivity of the outcome analysis among the different experimental models of brain injury, we calculated the minimum number of animals per group to evaluate the neurological, histological, and MRI outcomes between the models (Table 4). We also evaluated the sensitivity of the DABI model based on the mean and standard deviation data by calculating the minimum number of rats for the sham and DABI groups with a power of 0.8 alpha 0.05 for the NSS, MRI, and histological outcomes (see Table 3).

To our surprise, we found that the MRI method had greater sensitivity in identifying the DABI lesions compared to the histological methods. The results showed that in the fractional anisotropy and relative anisotropy protocol, the group size varied from five to ten rats per group (depending on the anatomical zone) compared with the histological method, which showed that the group size should be between six and fifteen rats (depending on the anatomical zone) (see Table 3). The radial diffusivity showed lower sensitivity compared to fractional anisotropy and relative anisotropy (see Table 3).

As expected, the neurological assessment also showed a high sensitivity for DABI lesions (see Table 3), which, as we previously noted, was the most sensitive parameter of brain injury in the TBI rat model [22].

This study has some limitations, including its small sample size. Although the rats were euthanized in this protocol, our results that confirm that MRI has greater sensitivity than histological results should promote more protocols that do not involve sacrificing animals in future studies. In addition, although we argue here that MRI is a more sensitive method than histology, this is not always the case. Here, specifically, our results show that the MRI method is sensitive enough to diagnose changes in the pathology of DABI.

By inducing DABI with a specialized model, we ensured that the pathologies that accompany TBI, such as edema, BBB destruction, inadequate blood supply to brain tissue, apoptosis, and other neurobiological events unrelated to DABI, did not influence the development of DABI, potentially complicating the study of the mechanism of this pathology and the testing of the approaches to its treatment.

## 4. Materials and Methods

The experiments were conducted in accordance with the recommendations of the Declarations of Helsinki and Tokyo and the Guidelines for the Use of Experimental Animals of the European Community. The experiments were approved by the Animal Care Committee of Ben-Gurion University in Negev, Israel.

### 4.1. Animals

The experiments were performed with a total of 20 male Sprague-Dawley rats (Harlan Laboratories, Jerusalem, Israel) weighing between 280 and 320 g each. Purina Chow and water were available ad libitum. The rats stayed in 12 h:12 h light-dark conditions at a constant temperature of 22 °C ± 1 °C. All experiments were performed in the dark phase between 08:00 and 16:00.

### 4.2. Experimental Design

**Figure 3:**
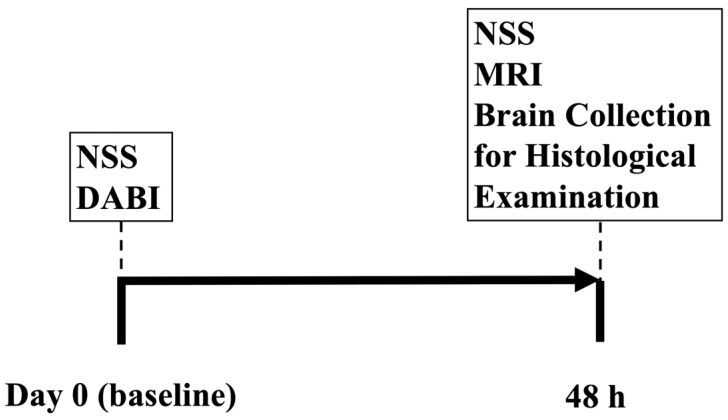
Experimental timeline. NSS: neurological severity score; DABI: diffuse axonal brain injury; MRI: magnetic resonance imaging.

A total of 20 rats were randomly allotted into one of 2 groups: naïve sham-operated rats (n = 10 rats) and moderate DABI (n = 10 rats). Neurological severity was evaluated at the baseline before the intervention and at 48 h following DABI (see Figure 3). Following the neurological evaluation at 48 h, all rats were scanned on a clinical MRI scanner and then euthanized for histological evaluation.

### 4.3. Neurological Severity Score (NSS)

The NSS was recorded by two individual blinded observers, as previously described [22,34]. Points were tallied based on the changes in motor functions and behavior, with the maximal score of 25 for the greatest neurological dysfunction and a score of 0 indicating an intact neurological condition. These parameters were considered: ability to exit from a circle (3-point scale), gait on a wide surface (3-point scale), gait on a narrow surface (4-point scale), effort to remain on a narrow surface (2-point scale), reflexes (5-point scale), seeking behavior (2-point scale), beam walking (3-point scale), and beam balance (3-point scale).

### 4.4. Induction of Moderate DABI

The apparatus was previously described by our team [36]. The device included four main mechanisms: (1) a transparent plastic cylinder; (2) an iron weight; (3) a rotation mechanism with a cylindrical tube, two bearings that the axis rotates on, and a head fixation (for ear pins); (4) a horizontal platform with two attached bearings.

The equation for calculating the rotational kinematics is as follows:

(1) $$M=\frac{2\times (K+mgl1)}{\pi }$$

(2) $$M=0.225m\times (1+\frac{14.2mh)}{1.664+1.037m})$$

(3) $$F_{o}=\frac{2.5}{D}m\times (1+\frac{14.2mh}{1.664+1.037m})$$

*F*_o_: force applied to the animal head (kg); *M*: moment of force; *K*: kinetic energy; m: mass of the falling weight; *g*: gravitational acceleration; *h*: height (cm); *D*: distance between the ear pins (cm). In our study, the force applied to the animal head was 2.3 kg.

The induction of DABI was performed as previously described [34]. Rats were anesthetized via the inhalation of isoflurane (5% for induction and 1.5–2.5% for maintenance) with the administration of equal parts medical air and oxygen. After induction of anesthesia, the rat’s head was fixed in the device, and an ear pin was inserted into the ipsilateral external auditory canal; a description of the method and videos can be found in a previously published protocol [34]. A free-falling weight was let go from a height of 120 cm to hit the bolt, activating the rotation mechanism. The lateral head rotation device caused the rodent’s head to turn rapidly from 0 to 90°. Following this procedure, the rat was awakened and transferred to a recovery room.

### 4.5. Histology

In order to measure the density of the β-APP-positive axons in the rats’ brains, immunohistochemistry staining was performed 48 h after the MRI procedure by a technician blinded to the groups. The rats’ chests were exposed, and the animals were perfused with cooled saline through the left ventricle at a pressure of 110 mm Hg until colorless perfusion fluid was collected from the right atrium, followed by 500 mL of 4% paraformaldehyde in 0.1 M phosphate buffer saline (pH 7.4). The brain was removed immediately and fixed in a 4% buffered formaldehyde solution for 48 h at 4 °C. The brain was then divided into 5 mm coronal slices from the olfactory bulb to the visual cortex. After paraffin embedding, slices of 5 μm were excised using microtome sectioning [34]. Immunochemical staining and examinations were performed as detailed previously [34]. Slices were placed on glass slides with a soft brush, with each slide holding 1 slice. The slices were deparaffinized with xylene and rehydrated with concentrations of ethanol at room temperature with the amount of ethanol reducing each time: 3 min in 100% ethanol twice, 3 min in 95% ethanol twice, 3 min in 90% ethanol, 3 min in 70% ethanol, and 3 min in deuterium-depleted water (DDW). Brain sections were treated with 3% H_2_O_2_ for 15 min at room temperature to block endogenous peroxidase activity and subsequently incubated with 0.01 M sodium citrate (pH 6.0) at 98 °C for 5 min for antigen retrieval. The slides remained in the buffer for 20 min at room temperature to reach a normal temperature, followed by washing with a phosphate-buffered saline (PBS) solution twice for 5 min. Sections were blocked with 2.5% normal horse serum for 1 h at room temperature and incubated overnight at 4 °C in primary rabbit anti-APP (1:4000) diluted in the blocking serum.

After incubation with the primary antibody, the sections were washed in PBS at room temperature. The sections were then incubated in appropriately diluted biotinylated secondary antibody for 15 min and washed with PBS for 3 min twice at room temperature. Next, the sections were incubated in streptavidin–peroxidase for 15 min, followed by another wash in PBS for 3 min twice at room temperature. Next, the sections were incubated with a buffered substrate solution (pH 7.5) containing hydrogen peroxide and a 3,3-diaminobenzidine chromogen solution and protected from light until the color developed. Finally, the slides were incubated with DDW at room temperature for 5 min in order to stop the reaction.

Next, the sections were counterstained with Hematoxylin for 3 min at room temperature and washed for 5 min with flowing tap water. The slides were then dehydrated with gradually increasing concentrations of ethanol at room temperature: 2 min in DDW, 2 min in 70% ethanol, 2 min in 90% ethanol, 2 min in ethanol 95%, 2 min in 100% ethanol, and 3 min in xylene three times. They were dried and mounted with mounting medium and examined under a microscope magnification of 200x with a 20 mm objective lens.

### 4.6. Diffusion-Weighted Imaging (DWI)

A 3-T MRI machine (Ingenia, Philips Medical Systems, Best, The Netherlands) with an eight-channel receive-only coil was used to perform DWI at 48 h following the intervention, as described previously [22]. The animals were maintained under general anesthesia (1.5% isoflurane in oxygen). Diffusion tensor imaging in 6 directions was conducted axially using a multi-shot, spin-echo, echo-planar sequence with TR/TE = 1419/138 msec and an epi factor of 19, a SENSE factor of 1.5, a b-factor of 1000 s/mm^2^, and spectrally selective fat suppression. Seven slices were acquired with zero gaps. The resolution (freq × phase × slice) was 0.55 × 0.55 × 2.0 mm. Five signal averages were collected for a scan time of 11:19 min. The Intellispace Portal workstation (V5.0.0.20030, Philips Medical Systems, Best, The Netherlands) was used for the post-processing of the permeability and perfusion studies.

### 4.7. Regions of Interest (ROI)

Images were collected to produce a non-linear transformation that combined each animal’s space to make a composite, averaged brain. On this brain, regions of interest (ROI) were designated by matching the new image with a custom-built 3D reconstruction of the Paxinos and Watson Rat Brain Atlas [36,37]. The ROI (left and right) were carefully delineated for the thalamus, hypothalamus, hippocampus, corpus callosum, and neocortex (see Figure 2). The values of the DTI metrics acquired from the locations of three consecutive imaging slices at bregma +1.0 mm, −1.0 mm, and −3 mm were averaged for each anatomical region.

### 4.8. DWI Parameter Map Analysis

An expert who was blind to the group assignments analyzed the images. Quantitative mean diffusivity (MD), axial diffusivity (AD), radial diffusivity (RD), relative anisotropy (RA), and fractional anisotropy (FA) maps in units of square millimeters per second were produced using the Philips software package version 5.7.1.2 (Ingenia, Philips Medical Systems, Best, The Netherlands). The values of the DTI metrics were averaged for the ROI [38] and subsequently analyzed [38,39].

### 4.9. Statistical Analysis

The statistical analysis was completed with the SPSS 22 package. A Kolmogorov–Smirnov test was performed to determine the appropriate test for the comparisons between the different parameters. The significance of the comparisons between the groups was determined using the Mann–Whitney, two-sided (for the non-parametric data; NSS) *t*-test, and two-sided tests (for the parametric data; MRI parameters and histology results). Prior to conducting the *t*-tests, the assumption of homogeneity of variances between the two groups was evaluated using Levene’s test for equality of variances. When Levene’s test indicated significant differences in variance between the groups (*p* < 0.05), indicating unequal variances, we adjusted the degrees of freedom for the subsequent *t*-tests according to the Welch–Satterthwaite equation. With a Pearson’s test, we calculated the correlation between the MRI parameters. The normally distributed data and continuous variables appeared as an average ± SD. Non-parametric data appeared as a median ± inner quartile range. The results were noted as statistically significant when *p* < 0.05.

## 5. Conclusions

The rotational acceleration-based DABI model is an appropriate and effective experimental model for studying diffuse axonal injury, allowing for an accurate assessment of the lesion using histology, neurology, and MRI techniques. We found that the most sensitive parameter for assessing lesions in the DABI model among histological, neuronal, and MRI outcomes was the MRI method, which is easily reproducible and less invasive than comparable brain analyses. Among the MRI protocols for assessing DABI, the most sensitive parameters are FA and RA, measured in the neocortex and thalamus, respectively. Among the other experimental models of brain injury, the MRI technique in the DABI model showed the greatest sensitivity compared with the TBI and stroke models. MRI is an accurate, highly sensitive, and highly specific method for assessing diffuse axial injury, particularly in the DABI model, and may be recommended at behavioral or other stages when animals cannot be euthanized, as when they need to be studied over an extended period of time. We anticipate that this protocol will assist with the study of DABI in a variety of contexts and hope that it will guide future research into treatment modalities for specific pathologies of brain injury.

## Figures and Tables

**Table 1:** Determination of the neurological severity scores 48 h after DABI. There was a significant difference in the NSS between the DABI group at 48 h after DABI and the sham-operated group, marked with a star (*p* < 0.01). The data were measured as counts and presented as medians and ranges.

NSS Values of the Study Groups		
Animal Groups	N	Median (Range)
Sham-operated	10	0(0–0)
DABI	10	1(1–2.5) *

**Table 2:** Correlation comparisons between the MRI parameters in the DABI rats. T: thalamus; Hy: hypothalamus; N: neocortex; Hi: hippocampus; CC: corpus callosum.

			MRI									
			Fractional Anisotropy					Radial Diffusivity				
			T	Hy	N	Hi	CC	T	Hy	N	Hi	CC
MRI	Relative Anisotropy	T	R = 0.97 *p* < 0.01	ns	ns	ns	ns	R = 0.87 *p* < 0.01	R = 0.67 *p* < 0.05	ns	ns	ns
		Hy	ns	R = 0.99 *p* < 0.01	ns	ns	ns	ns	R = 0.73 *p* < 0.05	R = 0.65 *p* < 0.05	ns	ns
		N	ns	R = 0.72 *p* < 0.05	R = 0.98 *p* < 0.01	ns	ns	ns	ns	R = 0.78 *p* < 0.01	ns	ns
		Hi	ns	ns	ns	R = 0.99 *p* < 0.01	ns	ns	ns	ns	ns	ns
		CC	ns	ns	ns	ns	R = 0.93 *p* < 0.01	ns	ns	ns	ns	ns
	Radial Diffusivity	T	R = −0.87 *p* < 0.01	ns	ns	ns						
		Hy	R = −0.65 *p* < 0.05	R = −0.76 *p* < 0.01	ns	ns						
		N	ns	R = 0.68 *p* < 0.05	R = 0.69 *p* < 0.05	ns						
		Hi	ns	ns	ns	ns						
		CC	ns	ns	ns	ns						

**Table 3:** Method to assess sensitivity. We calculated the sample size using the sample size calculator Version 1.061 for two equivalent groups (DABI and sham) based on a 95% confidence level (alpha = 0.01), mean ± SD, and a power of 80%. The data are expressed as a mean ± SD.

	Histological	MRI			NSS
		Fractional Anisotropy	Relative Anisotropy	Radial Diffusivity	
**Thalamus**	*n* = 6 20.5 ± 6.9 vs. 3.4 ± 5.3	*n* = 6 0.25 ± 0.08 vs. 0.47 ± 0.07	*n* = 5 0.22 ± 0.06 vs. 0.43 ± 0.07	*n* = 8 0.73 ± 0.1 vs. 0.54 ± 0.09	N = 8 1(1–2.5) vs. 0(0–0)
**Hypothalamus**	N = 6 32.5 ± 10.4 vs. 6.8 ± 10	N = 10 0.3 ± 0.05 vs. 0.39 ± 0.05	N = 10 0.26 ± 0.04 vs. 0.34 ± 0.05	n-s	
**Neocortex**	N = 15 22.6 ± 10 vs. 8.1 ± 11.6	N = 5 0.25 ± 0.04 vs. 0.39 ± 0.05	N = 6 0.21 ± 0.04 vs. 0.36 ± 0.07	N = 24 0.78 ± 0.16 vs. 0.64 ± 0.1	
**Hippocampus**	N = 13 8.3 ± 2 vs. 3.3 ± 4.3	N = 8 0.24 ± 0.05 vs. 0.38 ± 0.08	N = 7 0.2 ± 0.04 vs. 0.33 ± 0.07	N = 10 0.82 ± 0.09 vs. 0.68 ± 0.07	
**Corpus Callosum**	N = 9 19.8 ± 4.2 vs. 6.5 ± 8.5	N = 10 0.27 ± 0.02 vs. 0.41 ± 0.1	N = 9 0.23 ± 0.03 vs. 0.38 ± 0.1	N = 21 0.75 ± 0.11 vs. 0.65 ± 0.06	
**Calculated Sample Size per Group**	N = 6–15 rats per group	N = 5–10 rats per group	N = 5–10 rats per group	N = 8–21 rats per group	N = 8 rats per group

**Table 4:** Sensitivity analysis for different experimental models. We calculated the sample size using the sample size calculator Version 1.061 for two equivalent groups (injury and sham) based on a 95% confidence level (alpha = 0.01), mean ± SD, and a power of 80%. The data are expressed as a mean ± SD. Data for the stroke, SAH, and TBI models were taken from our earlier works [24,25,27,28,29,30]. We used the T2 protocol to assess brain edema in models of stroke and TBI. We used the T1 (Ktrans) protocol to assess BBB permeability in models of stroke and TBI. We used the DWI (ADC) protocol to assess lesion volume in models of stroke and TBI. We used the DWI (mean diffusivity, axial diffusivity, radial diffusivity, relative anisotropy, and fractional anisotropy) protocol to assess MRI outcomes in DABI models. IZ: infarct zone; BE: brain edema; BBB: blood–brain barrier; ADC: apparent diffusion coefficient; TBI: traumatic brain injury; SAH: subarachnoid hemorrhage; DABI: diffuse axonal brain injury; MRI: magnetic resonance imaging; NSS: neurological severity score.

Models of Brain Injury in Rats	Histological			MRI			NSS
	IZ	BE	BBB	ADC	T2	K_trans_	
Stroke	n = 10; 8.27 ± 5.9% vr. 0.31 ± 0.48%	n = 4; 12.5 ± 2.6% vr. 1.5 ± 2.3%	n = 5; 2352 ng/g ± 671 vs. 85 ng/g ± 26	n = 6; 5.98 ± 2.1% vr. 0.58 ± 0.3%	n = 10; 3.46 ± 2.1% vr. 0.66 ± 0.3%	n = 15; 2.01 ± 1.5% vr. 0.46 ± 0.3%	n = 4; 3(2–4) vr. 0(0–0)
TBI	n = 11; 4.4 ± 2.07% vr. 1 ± 2%	n = 13; 8.8 ± 6.5% vr. 1.3 ± 1.1%	n = 9; 5.0 × 10^−7^ g ± 2.5 × 10^−7^g vr. 1.2 × 10^−7^ g ± 0.5 × 10^−7^ g	n = 8; 2.63 ± 1.3% vr. 0.4 ± 0.5%	n = 12; 6.1 ± 3.8% vr. 0.7 ± 3.3%	n = 9; 5.8 ± 3.2% vr. 1 ± 0.5%	n = 7; 4(2–6) vr. 0(0–0)
SAH		n = 20; 79.32 ± 0.3% vr. 78.95 ± 0.36%	n = 22; 9.7 × 10^−7^ g ± 8.2 × 10^−7^ g vr. 3.1 × 10^−7^ g ± 1.8 × 10^−7^ g				n = 2; 9(0–10) vr. 0(0–0)
DABI	Immunohistochemistry for β-APP in the Thalamus			Fractional anisotropy in the neocortex	Relative anisotropy in the thalamus	Radial diffusivity in the thalamus	n = 8; 1(1–2.5) vs. 0(0–0)
	n = 6; 20.5 ± 6.9% vs. 3.4 ± 5.3%			n = 5; 0.25 ± 0.04% vs. 0.39 ± 0.05%	n = 5; 0.22 ± 0.06% vs. 0.43 ± 0.07%	n = 8; 0.73 ± 0.1% vs. 0.54 ± 0.09%	

## Data Availability

The data are available upon reasonable request.
